# Schizandrin A ameliorates cognitive functions via modulating microglial polarisation in Alzheimer’s disease mice

**DOI:** 10.1080/13880209.2021.1941132

**Published:** 2021-07-02

**Authors:** Qi Wang, Li Liu, Huibo Guan, Yanyan Zhou, Quan Li

**Affiliations:** a Teaching and Research Department of Basic Theory of Traditional Chinese Medicine, Heilongjiang University of Chinese Medicine, Harbin, Heilongjiang, China; b Department of Cardiovascular Diseases, First Affiliated Hospital, Heilongjiang University of Chinese Medicine, Harbin, Heilongjiang, China; c Hospital Office, First Affiliated Hospital, Heilongjiang University of Chinese Medicine, Harbin, Heilongjiang, China

**Keywords:** Microglial cells, apoptosis, cognitive disorder

## Abstract

**Context:**

Schizandrin A (Sch A) is a major phytochemical from *Schisandra chinensis* (Turcz.) Baill. (Schisandraceae), which exerts a neuroprotective effect in Alzheimer's disease (AD).

**Objective:**

To investigate the mechanism of Sch A in AD.

**Materials and methods:**

AD group: APP/PS1 transgenic mice served as AD models; AD + SCH group: APP/PS1 received 2 mg/kg Sch A by intragastric administration; WT: C57BL/6 mice were used as control. For *in vitro* assay, mouse microglial BV2 cells were treated with 0.5 µg/mL lipopolysaccharide or combined with 10 μmol/L Sch A for 24 h. The cognitive function and apoptosis in the mice was estimated. Microglial polarisation in the mice and cells was analysed.

**Results:**

Sch A treatment effectively improved spatial learning and memory ability and suppressed apoptosis in the brain tissues of APP/PS1 mice. APP/PS1 mice exhibited an increase in the levels of Aβ1-42 (2367.9 ± 431.1 pg/mg) and Aβ1-40 (1753.3 ± 253.4 pg/mg), which was abolished by Sch A treatment. Moreover, Sch A treatment repressed the proportions of iNOS^+^/Iba-1^+^ cells and IL-6 expression, while enhanced the proportions of Arg-1^+^/Iba-1^+^ cells and IL-10 expression in APP/PS1 mice. *In vitro*, Sch A treatment reduced the proportions of CD16/32^+^ cells, iNOS expression and IL-6 levels (25.7 ± 5.3 pg/mL) repressed M1 polarisation, and enhanced the proportions of CD206 cells, Arg-1 expression and IL-10 levels (75.9 ± 12.8 pg/mL) in BV2 cells.

**Conclusions:**

This research confirms the neuroprotective effect of Sch A in AD, suggesting that Sch A may become a potential anti-AD agent.

## Introduction

Alzheimer's disease (AD) is a neurodegenerative disease that characterized by loss of memory and cognitive decline. Its main clinical manifestations are progressive memory impairment, cognitive dysfunction, personality changes and language impairment (Lane et al. 2018). The pathological features of AD are mainly β-amyloid (Aβ) protein deposition to form senile plaques in the cerebral cortex and hippocampus, neurofibrillary tangles, neuron loss, decrease of choline acetyltransferase, and acetylcholine levels (Pimplikar et al. 2010). However, the aetiology and pathogenesis of AD have not yet been fully elaborated.

As the brain's innate immune cells, microglial cells play an irreplaceable role in the pathogenesis of AD (Hansen et al. 2018). On one hand, NF-κB and other transcription factors are activated upon the stimulation of Aβ, thereby causing the transcription of various inflammatory factors. Inflammatory factors such as IL-1β and TNF-α produced by microglial cells mediate inflammation (Katsumoto et al. 2018). On the other hand, microglial cells mediate the phagocytosis of Aβ and reduce Aβ deposition. Microglial cells can also form a microglia barrier through Trem2, and thus reduce the harm of Aβ to AD patients, and delay the development of AD (Condello et al. 2018). The dual roles of microglial cells in AD depend on the different polarization states of microglial cells. Microglial cells can be divided into classic activated (M1) phenotype and alternative activated (M2) phenotype. M1 microglial cells aggravate inflammatory response by secreting pro-inflammatory mediators including TNF-α, IL-1β, etc., thereby resulting in damage to the central nervous tissues. M2 microglial cells eliminate metabolic waste of central nervous system through the expression of scavenger receptor (CD206). Moreover, M2 microglial cells participate in repair of central nervous system injury and immune protection by secreting neuroprotective factors (Yao and Zu 2020).

The traditional Chinese medicine, *Schisandra chinensis* (Turcz.) Baill. (Schisandraceae), has effects on the central nervous system, such as improving learning, memory, sedation, hypnosis, anti-aging, and analgesia. Schizandrin A (Sch A) (C_24_H_32_O_7_) is a secondary metabolite extracted from the fruit of *S. chinensis*. Lyles et al. (2017) have confirmed the structure of Sch A. Sch A has a function in protecting liver injury through antioxidation, anti-inflammatory, and accelerating liver regeneration (Zhu et al. 2019). Research demonstrates the anticancer activities of Sch A in various cancers, such as breast cancer, colon carcinoma, and ovarian cancer (Kong et al. 2018; Lee et al. 2018; Zhang et al. 2018). Additionally, Sch A exerts neuroprotective effects in AD progression. Previous study has found that Sch A reduces cognitive disorder of AD mice by inhibiting neuroinflammation, oxidative stress, and endoplasmic reticulum stress (Qi et al. 2019; Song et al. 2020). Sch A inhibits Aβ-mediated neuron damage in AD by activating the PI3K/Akt signalling pathway, and reducing the expression of tau protein (Zhao et al. 2019). Previously, we found that Sch A suppressed NLRP1 inflammasome-mediated neuronal pyroptosis and neuronal apoptosis *in vivo* and *in vitro* (Li et al. 2021). However, whether Sch A can improve AD by regulating microglial polarisation is still unclear. Here, we investigate the mechanism of action of Sch A in regulating the polarisation of microglial cells in AD.

## Materials and methods

### Materials

Male APP/PS1 double transgenic mice (aged 3 months, weighting 24–30 g) and background-matched male C57BL/6 wide-type (WT) mice (aged 3 months, weighting 24–30 g) were obtained from the Model Animal Research Centre of Nanjing University (Nanjing, China). Mouse microglial BV2 cells were derived from CCTCC (Wuhan, China). Sch A (98% purity) was purchased from Weikeqi Biotech (Sichuan, China). Mouse Aβ1-40 ELISA Kit, Mouse Aβ1-42 ELISA Kit, Mouse IL-6 ELISA Kit and Mouse IL-10 ELISA Kit were purchased from Mlbio (Shanghai, China). Dulbecco’s modified Eagle’s medium (DMEM) and foetal bovine serum (FBS) were obtained from Gibco (Rockville, MD, USA). TUNEL apoptosis assay kit and Penicillin-Streptomycin Liquid were derived from Solarbio (Beijing, China). TRIzol reagent was purchased from Invitrogen (Carlsbad, CA, USA). The antibodies, Iba-1, iNOS, Arg-1, CD16/32-FITC, CD206-FITC and HRP-IgG, were obtained from Proteintech (Wuhan, China). PrimeScript™ RT reagent Kit and TB Green® Premix Ex Taq^™^ II (Tli RNaseH Plus) were purchased from Takara (Tokyo, Japan).

### Animals

APP/PS1 and C57BL/6 mice were housed under specific pathogen free condition with a 12-h light/dark cycle at 20–24 °C. The mice accessed to food and water freely. All protocols were authorised by the Ethics Committee of the First Affiliated Hospital of Heilongjiang University of Chinese Medicine.

Mice were randomly divided into three groups (*n* = 10): (1) AD + Sch A group: APP/PS1 mice received Sch A (2 mg/kg, dissolved in 0.1% DMSO) by intragastric administration once a day for 2 weeks (Wei et al. 2018). (2) AD group: APP/PS1 mice received the same volume of distilled water by intragastric administration once a day for 2 weeks. (3) WT group: C57BL/6 WT mice received the same volume of distilled water as a control.

After 2 weeks of Sch A or distilled water administration, the spatial learning and memory ability of mice were detected by Morris water maze. Then, the mice were euthanized by cervical dislocation. The brain tissues of mice were rapidly collected and stored at −80 °C, or fixed in 4% paraformaldehyde and embedded in paraffin for further experiments.

### Morris water maze

The spatial learning and memory ability of APP/PS1 and C57BL/6 mice were detected by Morris water maze as previous reported (Wang et al. 2019). Briefly, the water maze was a circular pool (122 cm in diameter) filled with water (30 cm deep, at 25 ± 1 °C). The maze was divided into four equal quadrants based on four directions: NE, NW, SW, and SE. There was a visible escape platform (12 cm in diameter) located in the midpoint of fixed target quadrant 2 cm beneath the water surface. Morris water maze test was contained spatial training and probe test. For spatial training, mice were allowed to swim to find the hidden platform within 60 s, and these mice were required to stay on the platform for 10 s. If it failed, the mice were guided to the hidden platform and stayed on the platform for 10 s. After that, the mice were returned to their home cage. Each mouse was received for trails per day for 5 days in the spatial training. On the 6th day, the platform was removed, and the mouse was released in the opposite quadrant of the target quadrant. The mice were allowed to swim freely in the maze within 60 s. The latency period to find the hidden platform (escape latency), the distance travelled (path length), the percentage of time spent in the target quadrant, and the frequency of crossing the platform in each trail were recorded and analysed using Videomex tracking system (Columbus Instruments, Columbus, OH, USA).

### Enzyme-linked immunosorbent assay (ELISA)

The concentration of Aβ1-40 and in brain tissues of APP/PS1 and C57BL/6 mice, IL-6 and IL-10 in BV2 cells were measured by ELISA using Mouse Aβ1-40 ELISA Kit, Mouse Aβ1-42 ELISA Kit, Mouse IL-6 ELISA Kit and Mouse IL-10 ELISA Kit. All ELISA was carried out according to the protocols of manufacturer.

### TdT-mediated dUTP nick-end labelling (TUNEL) assay

TUNEL assay was performed to detect cell apoptosis in brain tissues of APP/PS1 and C57BL/6 mice. Brain tissues of mice were fixed in 4% paraformaldehyde and embedded in paraffin. After deparaffin and rehydration, 5 μm sections were obtained. Then, paraffin sections were stained applying TUNEL apoptosis assay kit according to the manufacturer’s protocol. The stained sections were then observed under an optical microscope (Nikon, Tokyo, Japan) at 200 × and 400 × magnification.

### Immunohistochemistry

Immunohistochemistry was performed to estimate the proportions of Iba-1^+^, iNOS^+^ and Arg-1^+^ microglial cells in APP/PS1 and C57BL/6 mice as the previous reported (Wang et al. 2019). Paraffin-embedded brain tissues were cut into 5 μm thick sections using a microtome. After deparaffin and rehydration, the sections were treated with fresh 3% H_2_O_2_ for 25 min at room temperature to eliminate the activity of endogenous peroxidases. After washing with PBS several times, the sections were blocked with 3% bovine serum albumin for 30 min and then incubated with primary antibodies, Iba-1 (1:1000), iNOS (1:500) or Arg-1 (1:500) at 4 °C overnight. Subsequently, the sections were incubated with HRP-IgG at room temperature for 30 min. Finally, the sections were washed with PBS, and then stained with diaminobenzidine and counterstained with haematoxylin. The sections were observed under an optical microscope (Nikon, Tokyo, Japan). Five different visual fields were selected in each section to count Iba-1-, iNOS- and Arg-1-positive cells.

### Cell culture

Mouse microglial BV2 cells were cultured in DMEM supplemented with 10% FBS and 1% penicillin/streptomycin at 37 °C in a 5% CO_2_/95% air incubator. BV2 cells were treated with 0.5 µg/mL lipopolysaccharide (LPS) for 24 h. Then, LPS-treated BV2 cells were treated with 10 μmol/L of Sch A (dissolved in 0.1% DMSO) for 24 h (Zhao et al. 2019). Normal BV2 cells served as a control.

### Flow cytometry

Flow cytometry was performed to examine the proportions of M1 and M2 microglial cells in BV2 cells. BV2 cells (1 × 10^7^ cells/mL, 100 μL) were incubated with 10 μL antibodies, CD16/32-FITC or CD206-FITC at room temperature for 30 min in dark. After washed with PBS, the proportions of M1 and M2 microglial cells in BV2 cells were detected using a FACSCalibur (BD Biosciences, San Jose, CA, USA). Furthermore, CD16/32 was used to mark M1 microglial cells. CD206 was used to mark M2 microglial cells.

### Quantitative real-time PCR (qRT-PCR)

QRT-PCR was performed to detect the expression of genes in brain tissues of APP/PS1 and C57BL/6 mice and BV2 cells. Total RNA extraction was carried out using TRIzol reagent. 1.5% Agarose gel electrophoresis was performed to assess the integrity of RNA. Total-RNA (1.0 µg) was used to synthesise complementary DNA applying PrimeScript^™^ RT reagent Kit. Gene expression was estimated by performing qRT-PCR using TB Green^®^ Premix Ex Taq^™^ II (Tli RNaseH Plus). The relative expression of genes was normalised to the expression of GADPH. The results were analysed using 2^-ΔΔCT^ method for quantification.

### Statistical analysis

All assays were carried out more than 3 times. Data were reported as mean ± standard deviation. SPSS 22.0 statistical software (IBM, Armonk, NY, USA) was used for statistical analysis. Two-tailed Student’s *t*-test and one-way ANOVA were used to analyse the statistical difference. Values of *p* < 0.05 were considered to be statistically significant.

## Results

### Sch A improved spatial learning and memory ability and reduced the levels of Aβ1-42 and Aβ1-40 in Alzheimer’s disease mice

**Figure 1.**
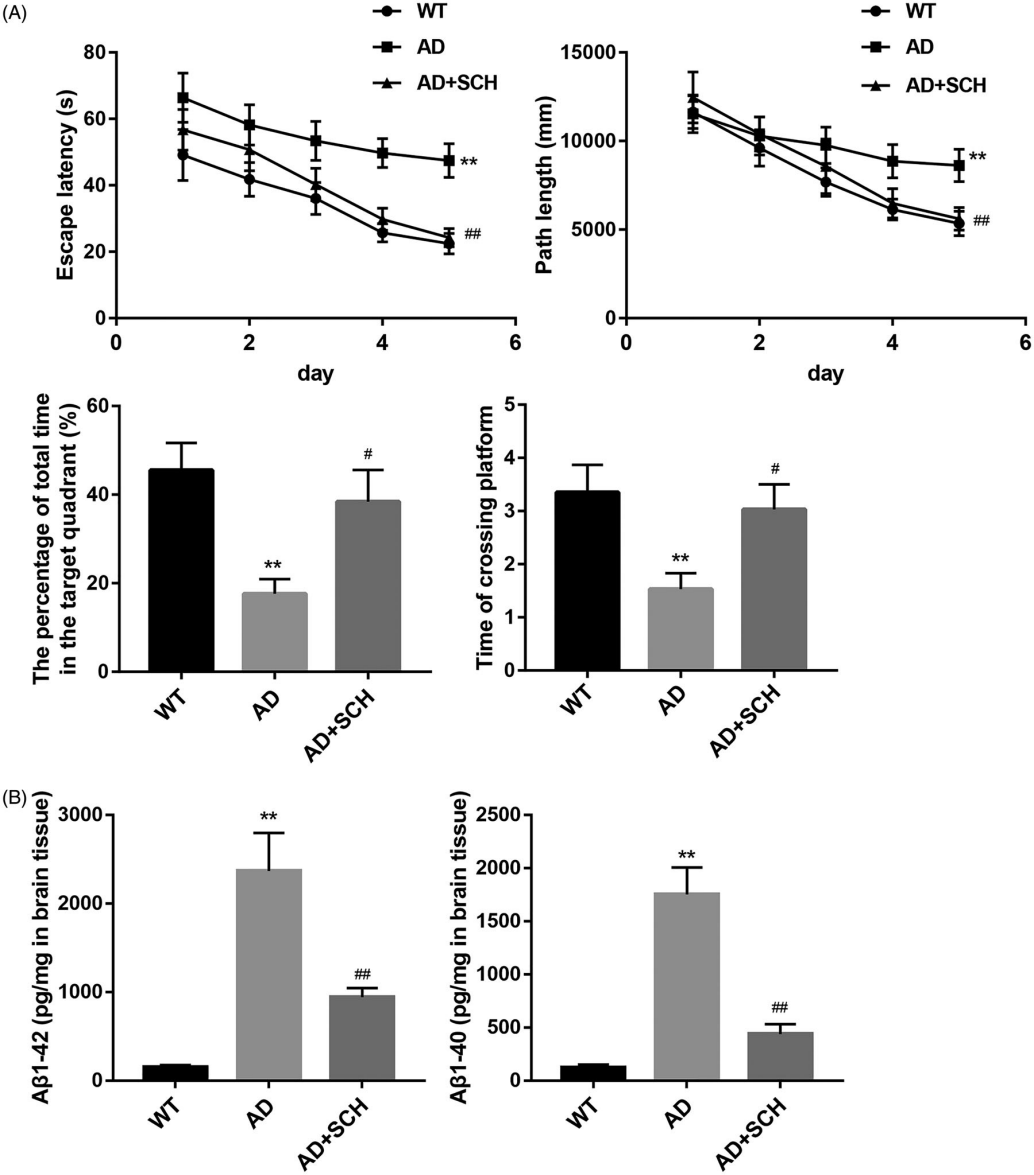
Schizandrin improved spatial learning and memory ability and reduced the levels of Aβ1-42 and Aβ1-40 in Alzheimer’s disease mice. APP/PS1 mice received Sch A or distilled water administration. C57BL/6 WT mice served as control. Morris water maze test was performed to analyse the escape latency (A), path length (B), the percentage of total time in the target quadrant (C) and the time of crossing platform (D) of mice. (E,F) The levels of Aβ1-42 and Aβ1-40 in the brain tissues of mice were estimated by ELISA. **p < 0.01 vs. WT; ^#^*p* < 0.05, ^##^*p* < 0.01 vs. AD.

We initially determined the influence of Sch A administration on the spatial learning and memory ability of AD mice by Morris water maze. Compared with WT mice, AD mice had a longer escape latency and path length. However, Sch A treatment effectively shortened the escape latency and path length of AD mice. AD mice spent less time in the target quadrant, whereas AD mice spent longer time in the target quadrant after Sch A treatment. The time of crossing platform was reduced in AD mice, which was abolished by Sch A treatment (Figure 1(A)). Furthermore, ELISA data showed that the levels of Aβ1-42 (2367.9 ± 431.1 pg/mg) and Aβ1-40 (1753.3 ± 253.35 pg/mg) were significantly enhanced in the brain tissues of AD mice as compared with the levels of Aβ1-42 (151.3 ± 24.47 pg/mg) and Aβ1-40 (124.8 ± 25.46 pg/mg) in WT mice. Sch A treatment effectively reduced the levels of Aβ1-42 (944.8 ± 102.26 pg/mg) and Aβ1-40 (438.8 ± 94.11 pg/mg) in the brain tissues of AD mice (Figure 1(B)). Therefore, Sch A improved the spatial learning and memory ability and reduced the levels of Aβ1-42 and Aβ1-40 in AD mice.

### Sch A inhibited cell apoptosis in brain tissues of Alzheimer’s disease mice

**Figure 2.**
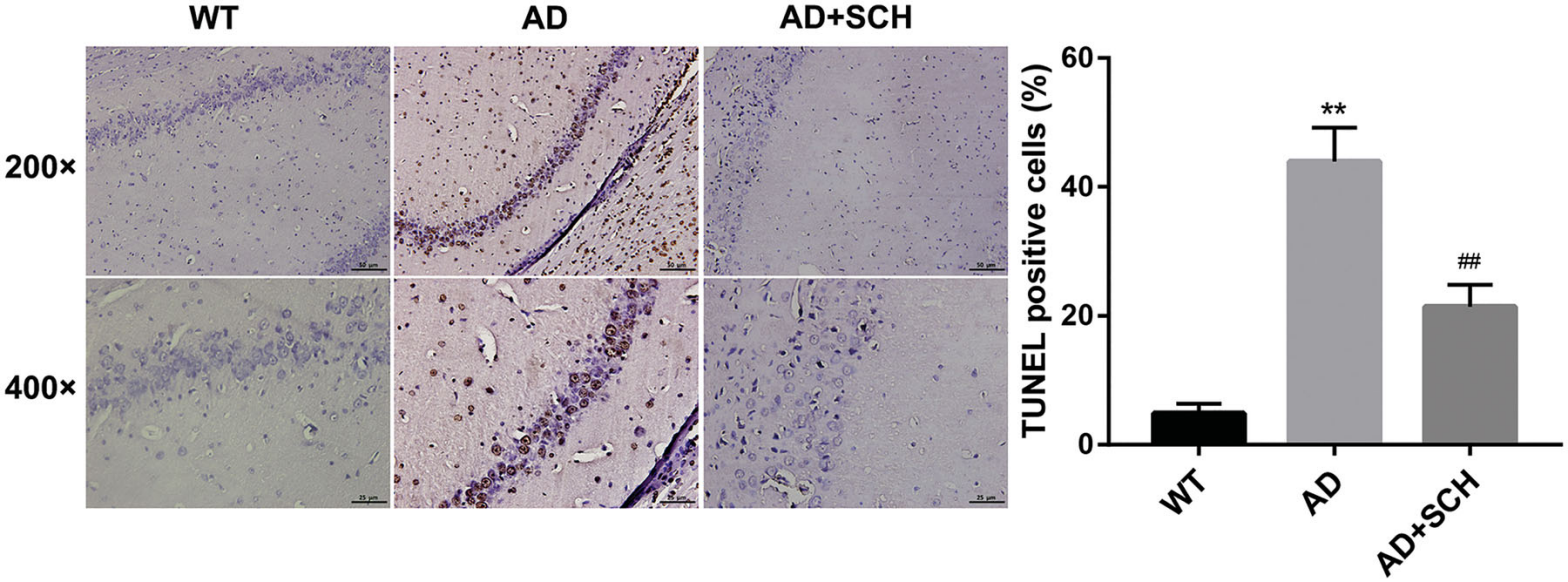
Schizandrin inhibited cell apoptosis in brain tissues of Alzheimer’s disease mice. APP/PS1 mice received Sch A or distilled water administration. C57BL/6 WT mice served as control. TUNEL assay was performed to assess cell apoptosis in brain tissues of mice. **p < 0.01 vs. WT; ^##^*p* < 0.01 vs. AD.

Next, we further examined the influence of Sch A treatment on cell apoptosis in brain tissues of AD mice by TUNEL assay. As shown in Figure 2, the brain tissues of AD exhibited a boost in apoptotic cells with respect to WT mice. Compared with AD mice, Sch A treatment repressed cell apoptosis in the brain tissues of AD mice (Figure 2).

### Sch A treatment inhibited M1 microglial polarization and promoted M2 microglial polarization in vivo

**Figure 3.**
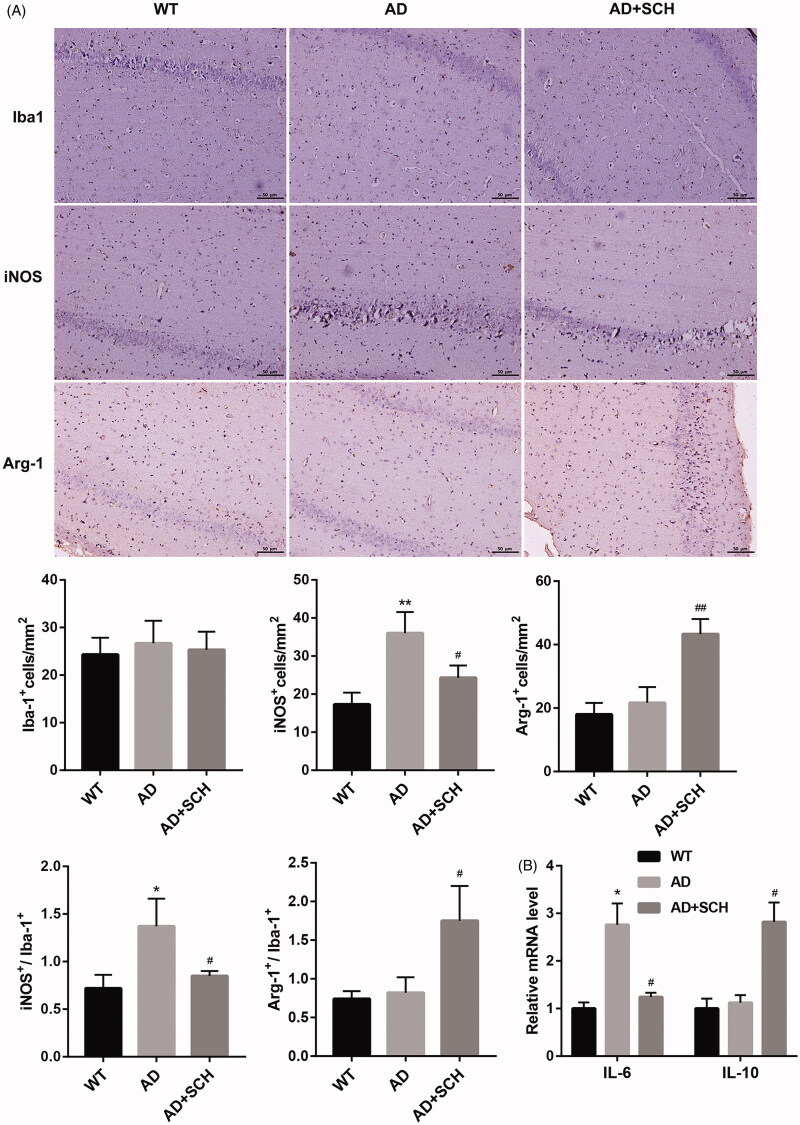
Schizandrin treatment inhibited M1 microglial polarisation and promoted M2 microglial polarisation in Alzheimer’s disease mice. APP/PS1 mice received Sch A or distilled water administration. C57BL/6 WT mice served as control. (A) The proportions of Iba-1^+^, iNOS^+^ and Arg-1^+^ microglial cells in brain tissues of mice was assessed by immunohistochemistry. (B) The expression of IL-6 and IL-10 in the brain tissues of mice was detected by qRT-PCR. **p < 0.01 vs. WT; ^##^*p* < 0.01 vs. AD.

In order to determine whether Sch A treatment improved AD in mice by regulating microglial proliferation, we stained the brain tissues of mice with the markers of M1 (iNOS), M2 (Arg-1) and microglial cells (Iba-1). The results of immunohistochemistry staining showed that there was no significant difference in the proportions of Iba-1-positive microglia cells among WT, AD and AD + Sch A groups. Compared with WT mice, the proportions of iNOS-positive microglial cells were increased in brain tissues of AD mice. The proportions of iNOS-positive microglial cells were decreased in AD mice following Sch A treatment. There was no difference in the proportions of Arg-1-positive microglial cells between WT and AD groups. Sch A treatment caused an increase in the proportions of Arg-1-positive microglial cells in AD mice. Moreover, AD mice exhibited an increase in the ratio of iNOS^+^/Iba-1^+^ microglial cells. However, the ratio of Arg-1^+^ to Iba-1^+^ microglial cells was not significantly different between WT and AD groups. Sch A treatment inhibited the ratio of iNOS^+^/Iba-1^+^ microglial cells and enhanced the ratio of Arg-1^+^/Iba-1^+^ microglial cells in AD mice (Figure 3(A)). We also found that AD exhibited a boost in the levels of IL-6 with respect to WT mice. Sch A treatment caused a decrease in the levels of M1 microglial cell marker IL-6, and an increase in the levels of M2 microglial cell marker IL-10. The levels of IL-10 were not significantly different between WT and AD groups (Figure 3(B)). Thus, these data demonstrated that Sch A treatment inhibited M1 microglial polarisation and promoted M2 microglial polarisation *in vivo*.

### Sch A treatment inhibited M1 microglial polarization and promoted M2 microglial polarization *in vitro*

**Figure 4.**
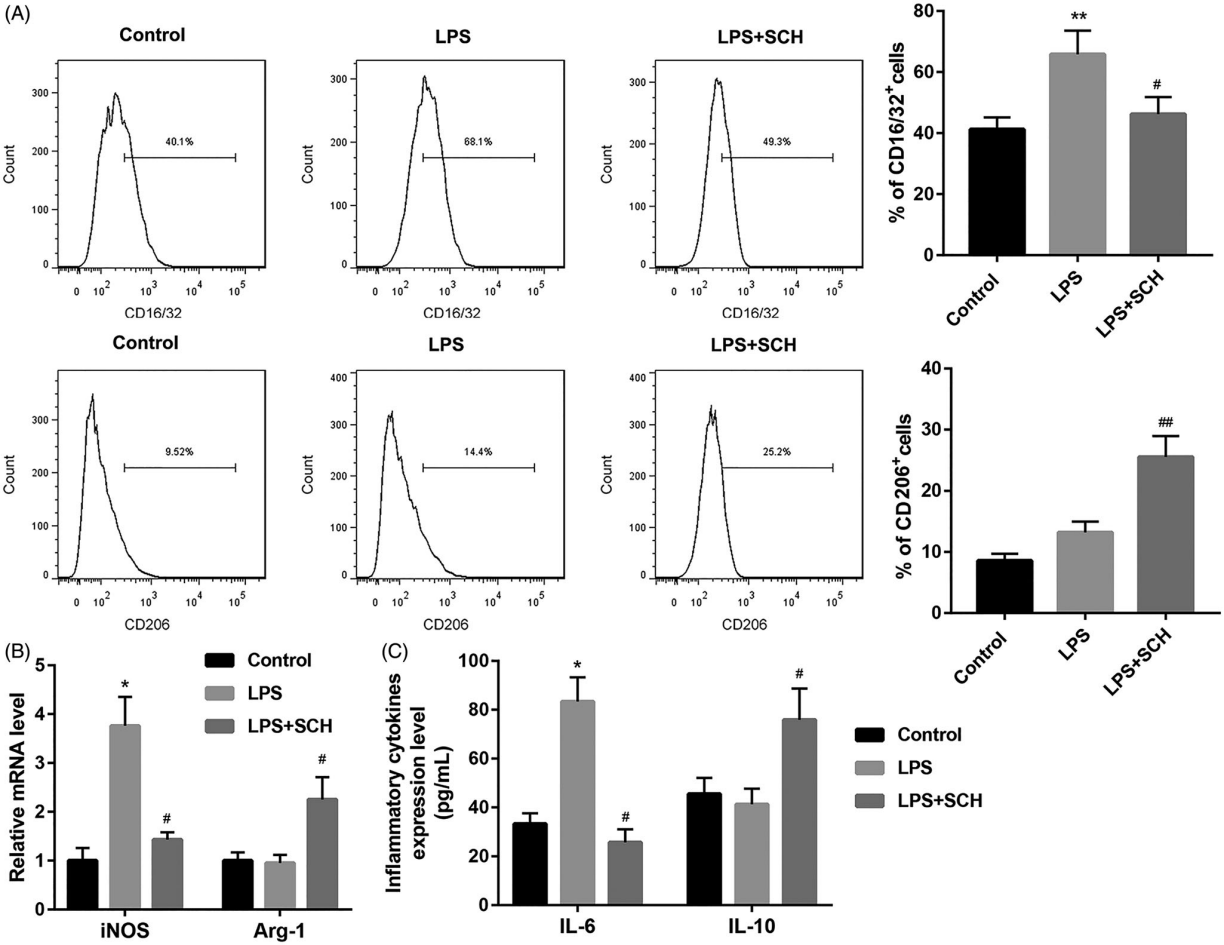
Schizandrin treatment inhibited M1 microglial polarisation and promoted M2 microglial polarisation in BV2 cells. BV2 cells were treated with LPS or combined with Sch A. Normal BV2 cells served as control. (A) Flow cytometry was performed to assess the proportions of M1 and M2 microglia in the BV2 cells. (B) The expression of iNOS and Arg-1 in the BV2 cells was detected by qRT-PCR. (C) The levels of IL-6 and IL-10 in the BV2 cells were estimated by ELISA. *p < 0.05, **p < 0.01 vs. Control, ^#^*p* < 0.05, ^##^*p* < 0.01 vs. LPS.

Finally, we determined the influence of Sch A on microglial polarization *in vitro*. The results of flow cytometry revealed that LPS treatment enhanced the proportions of CD16/32-positive cells in BV2 cells, which was abolished by Sch A treatment. LPS treatment had on influence on the proportions of CD206-positive cells in BV2 cells. However, the proportions of CD206-positive cells were enhanced in the LPS-treated BV2 cells following Sch A treatment (Figure 4(A)). Moreover, we performed qRT-PCR to examine the expression of iNOS and Arg-1 in the BV2 cells. LPS treatment significantly enhanced the expression of iNOS, whereas had no effect on Arg-1 expression in the BV2 cells. Sch A treatment caused a down-regulation of iNOS, while led to an up-regulation of Arg-1 in LPS-treated BV2 cells (Figure 4(B)). In addition, ELISA data showed that IL-6 levels (83.4 ± 9.9 pg/mL) were increased in LPS-treated BV2 cells as compared with control group (33.3 ± 4.3 pg/mL), whereas IL-10 levels were not significantly different between control (45.6 ± 6.6 pg/mL) and LPS-treated BV2 cells (41.3 ± 6.4 pg/mL). IL-6 levels (25.7 ± 5.3 pg/mL) were reduced and IL-10 levels (75.9 ± 12.8 pg/mL) were significantly enhanced in the LPS-treated BV2 cells in the presence of Sch A (Figure 4(C)). Taken together, these findings suggested that Sch A treatment inhibited M1 microglial polarisation and promoted M2 microglial polarisation *in vitro*.

## Discussion

Many Chinese patent medicines containing *S. chinensis* have been used in the treatment of various diseases, such as Shengmai and Wuzhi capsule. For example, Shengmai exerts a positive effect on heart failure and chronic obstructive pulmonary disease (Zhou et al. 2014; Huang et al. 2019). Wuzhi capsule has a efficacy in treating non-alcoholic fatty liver disease, the underlying mechanism likely involves the PPAR-α/γ and NF-κB signalling pathways (Chen et al. 2019). Additionally, *S. chinensis* is also as a dietary supplement contained in various multi-ingredient products sold in the United States (Lyles et al. 2017). The dietary supplement containing *S. chinensis*, Swedish Herbal Institute Adapt-232, can effectively improve the attention and cognitive accuracy of depression patients (Aslanyan et al. 2010). Adjuvant therapy with Chisan® (Adapt-232) has a positive effect on the recovery of patients with acute non-specific pneumonia (Narimanian et al. 2005). A plant preparation AdMax containing *S. chinensis* prevents chemotherapy-induced immunosuppression in patients with ovarian cancer (Kormosh et al. 2006). Moreover, accumulation researches have determined the biological role of Sch A in the progression of AD. For example, Sch A exerts a protective effect on AD rats by ameliorating cognitive impairments, endoplasmic reticulum stress, and neuroinflammation (Hu et al. 2019). Sch A combined with nootkatone coordinately inhibits inflammation, apoptosis, and autophagy through PI3K/AKT signalling pathway, thus exerts a neuroprotective role in AD progression (Qi et al. 2020). Wei et al. (2018) have confirmed that Sch A can regulate the levels of neurotransmitters and their metabolites in the brain tissues, thereby reducing Aβ deposition and improving the learning and memory ability in APP/PS1 mice. Consistently, the present study also found the protective effect of Sch A on AD mice. Sch A treatment effectively improved spatial learning and memory ability and repressed cell apoptosis in the brain tissues in AD mice. The levels of Aβ1-42 and Aβ1-40 were reduced in AD mice following Sch A treatment. Moreover, we focussed on exploring whether Sch A can improve AD by regulating microglial polarisation.

Microglial cells are widely involved in the progression of AD. The study of Liu et al. (2017) has found that Schizandrin B suppresses the levels of inflammatory factors such as TNF-α, IL-6, IL-1β, and PGE by regulating the activation of PPAR-γ, thereby exerting an anti-inflammatory effect on LPS-induced BV2 cells. Sch A inhibits neuroinflammation injury of LPS-induced BV2 cells and primary microglial cells through inhibition of the TRAF6-IKKβ-NF-κB and Jak2-Stat3 signalling pathways (Song et al. 2016). Schizandrin C promotes phase II detoxifying/antioxidant enzymes through cAMP/PKA/CREB and Nrf-2 signalling pathways, and thus attenuates the neuroinflammatory response in microglial cells (Park et al. 2013). Our study determined the biological role of Sch A in microglial polarisation in AD. Sch A treatment reduced the ratio of iNOS^+^/Iba-1^+^ microglial cells and enhanced the ratio of Arg-1^+^/Iba-1^+^ microglial cells in AD mice. Moreover, Sch A treatment repressed the levels of M1 microglial cell marker IL-6 and promoted the levels of M2 microglial cell marker IL-10 in AD mice. M1 microglial cells express iNOS, and M2 microglial cells express Arg-1 (Aryanpour et al. 2017). Thus, these data demonstrated that Sch A treatment inhibited M1 microglial polarisation and promoted M2 microglial polarisation in AD mice.

Microglial polarisation is closely associated with the progression of AD. M1 microglial cells accelerate inflammatory injury, while M2 microglial cells exert a neuroprotective effect in AD. Imbalanced M1 and M2 microglial polarisation markedly promotes the development of AD (Yao and Zu 2020). Xu et al. (2019) have confirmed that cGAMP promotes microglial polarisation from M1 towards M2 phenotype by promoting TREM2 expression, and thus ameliorates cognitive impairment, reduces Aβ deposition and neuron apoptosis in AD mice. M2 microglial cells effectively attenuates AD-related behaviour impairment and neuropathic pain (Jin et al. 2020). Chitinase1 has been reported to polarise microglial cells into an M2 phenotype, thereby reducing Aβ oligomer deposition and exerting protective effects against AD (Xiao et al. 2017). In our study, we found that Sch A polarised microglial cells into an M2 phenotype in AD mice and LPS-treated BV2 cells, and then mediating the progression of AD. Thus, this study initially revealed that Sch A suppressed AD development by affecting microglial polarisation. However, the present study initially revealed that Sch A regulated AD progression through regulating microglia polarisation. The underlying molecular mechanism of Sch A regulating the polarisation of microglia, and the polarisation of microglia involved in the process of AD have not been studied in depth. This is the shortcomings of this article. Thus, we will delve into the mechanism of Sch A in regulating microglia polarisation, and the function of Sch A in other neurodegenerative diseases such as Parkinson's disease.

This research confirms the neuroprotective effect of Sch A in AD. Sch A ameliorates cognitive functions via modulating microglial polarisation in Alzheimer’s disease mice. Thus, this study suggests that the Chinese patent medicine containing Sch A may become a potential anti-Alzheimer’s disease agent.
